# An Insight into the Molecular Electronic Structure of Graphene Oxides and Their Interactions with Molecules of Different Polarities Using Quantum Chemical and COSMO-RS Calculations

**DOI:** 10.3390/molecules29163839

**Published:** 2024-08-13

**Authors:** Víctor R. Ferro, Sonia Merino, Rafael Lopez, José L. Valverde

**Affiliations:** 1 Departamento de Ingeniería Química, Universidad Autónoma de Madrid, 28049 Madrid, Spain; 2 Departamento de Ingeniería Química, Universidad de Castilla la Mancha, 13091 Ciudad Real, Spain; soniamefe@gmail.com (S.M.); joseluis.valverde@uclm.es (J.L.V.); 3 Departamento de Química Física Aplicada, Universidad Autónoma de Madrid, 28049 Madrid, Spain; rafael.lopez@uam.es

**Keywords:** graphene oxides, molecular electronic structure, quantum chemical calculations, COSMO-based analyses, molecular interactions

## Abstract

A systematic theoretical study on the molecular electronic structure of graphene and its oxides, including their interactions with molecular species of different polarity, was carried out. The influence of the O/C atomic ratio in the graphene oxides was also evaluated. Quantum chemical and COSMO-based statistical-thermodynamic calculations were performed. Geometry optimizations demonstrated that graphene sheets are structurally distorted by oxygen substitution, although they show high resistance to deformation. Furthermore, under axial O-C bonding, proton-donor and proton-acceptor centers are created on the graphene oxide surface, which could acquire an amphoteric character. In low-oxidized graphene oxides, H-bonding centers coexist with neutral highly polarizable π electron clouds. Deep graphene oxidation is also related to the formation of a *quasi*-two-dimensional H-bond network. These two phenomena are responsible for the exceptional adsorption and catalytic properties and the potential proton conductivity of graphene oxides. The current calculations demonstrated that the interactions of polar molecular species with deep-oxidized graphene derivatives are thermodynamically favorable, but not with low-oxidized ones. The capacity of the quantum chemical and COSMO-RS calculations to model all these issues opens the possibility of selecting or designing graphene-based materials with optimized properties for specific applications. Also, they are valuable in selecting/designing solvents with good exfoliant properties with respect to certain graphene derivatives.

## 1. Introduction

In recent years, graphene-based materials have attracted the attention of the scientific and technological communities because their exceptional properties ensure interesting potential applications in different fields [1,2,3,4], such as electronics and nano-electronics, composite materials, spintronic devices, electromechanical resonators, quantum computing, impermeable atomic membranes, sensors, biomedicine, adsorbents, catalysts, etc. Graphene oxide (GO) is a peculiar material composed of single oxygen-functionalized graphene layers with a crystal structure like pristine graphite but more disordered and showing larger inter-sheet distances [5,6,7,8].

Some synthesis methods have been developed to produce GOs [2,7,9,10,11,12,13]; however, only those methods based on the oxidative exfoliation of graphite materials (the Hummers methods) result in high yields of GO and have succeeded to be reproducible, scalable, and techno-economically viable [11,12,13,14]. The selection of efficient exfoliants is a crucial aspect of these procedures. In addition, the response of these materials in the evaluated applications strongly depends on their intrinsic molecular electronic structure (applications in electronic devices, for example) and the interaction capacity of the surface (when they are used as adsorbent, catalyst, etc.). A wide set of experimental [15,16,17,18,19,20,21,22] and theoretical [8,22,23,24,25,26,27,28,29,30,31,32,33,34] techniques have been used to study the molecular electronic structure of the GOs, to characterize their surface, and to evaluate their interaction capacity with molecular species of different polarity. 

Among the theoretical methods, those based on the quantum chemical Density Functional Theory (DFT) have been popularized because they combine relatively low computational costs with both reasonable accurate results and predictive capacity. These methods include two main formalisms: (i) Graphene-based materials are considered extended periodic solid structures, and the calculations are carried out using pseudopotentials for the core electrons and plane-wave basis sets of the valence electrons [23,24,25,26,27,29]. This is recognized as the solid-state approach, based on the “first principle” closed-shell DFT approximation complemented by artificial periodic boundary conditions. This strategy is useful for describing the electronic structure (in terms of density of states around the Fermi energy, for example) of the pure solid, doped by heteroatoms or chemically modified. (ii) Graphene and graphene derivatives are modeled as *molecules* or *molecular* clusters using the self-consistent field molecular orbital (SCF-MO) method within the DFT theory. In the current approach, the interaction of the solid surface with molecules can be described as non-covalent aggregates formed by H-bonding, coulomb, and van der Waals interactions. In this way, for instance, catalytic and adsorption phenomena are frequently studied [22,28,30,31,32,34]. 

Using the second formalism mentioned above opens the possibility of applying the COSMO-based theories [35,36,37] to assess the intermolecular interactions incorporating quantum chemical and thermodynamic-statistic descriptors. This procedure formally requires considering the solid as a *pseudo*-liquid [38] and raises the issue that the dimension of the cluster constructed for modeling the solid should be big enough to correctly describe the phenomena under study. 

In the current work, considering the graphene and its oxides as discrete *molecules*, SCF-MO DFT and COSMO-based calculations were carried out to understand the consequences of the oxidation degree on both their molecular electronic structure and the interaction with molecules of different polarities. By convention, the words *molecule* and *molecular* are written in italics throughout the manuscript when they refer to the graphene and its oxides because they are treated as discrete molecules for the reasons explained above. 

## 2. Results and Discussions

### 2.1. Molecular and Electronic Structure of the GOs

**Figure 1:**
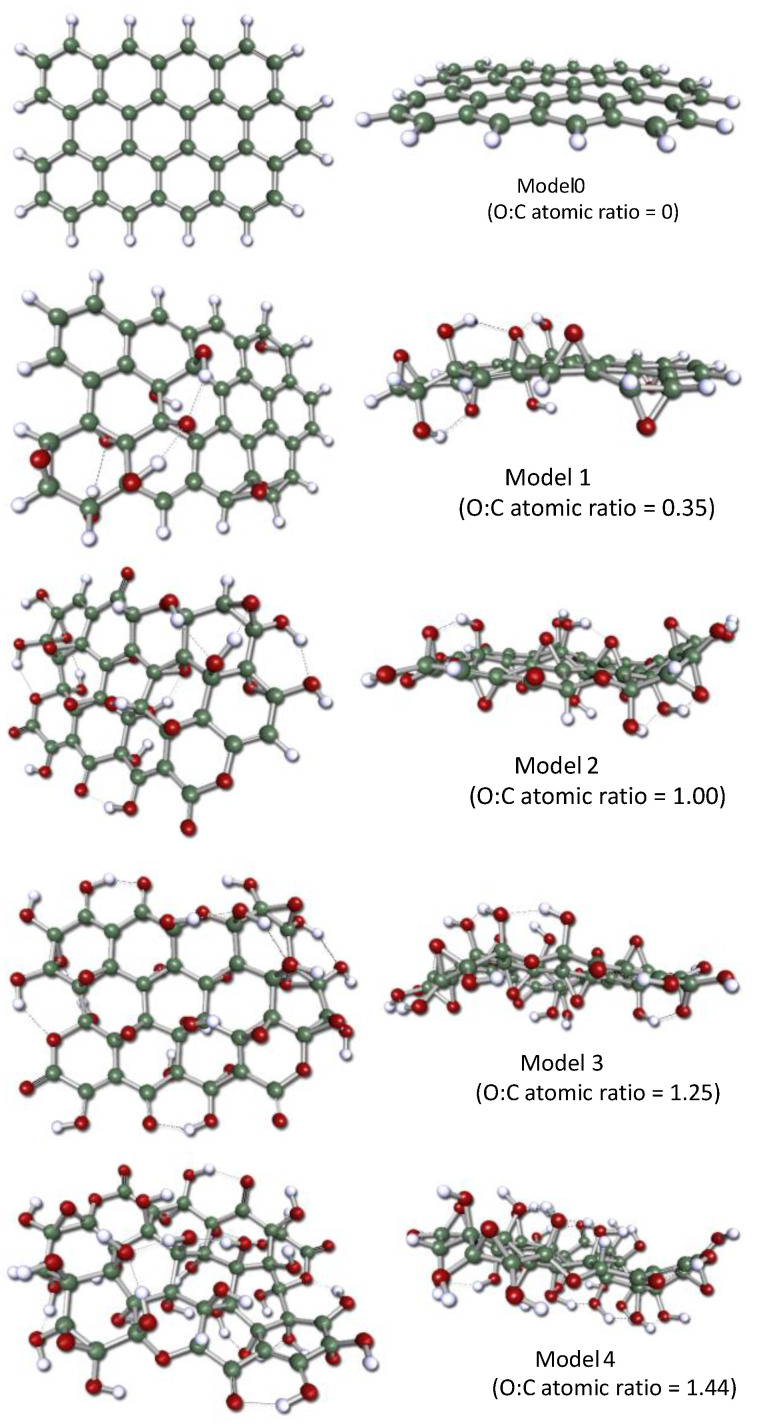
Optimized geometries (at the B3LYP/6-31G(d,p)) computational level) of the model structures used in this work to represent graphene (Model 0) and graphene oxides (Models 1 to 4) with different oxidation degrees. H-bonds, represented by dashed lines, were computed for d_(O···H)_ < 2.0 Å and a_(H···O-C)_ ≤ 120°. The atom color code: dark grey—carbon, red—oxygen, white—hydrogen. This code will be maintained in the remaining figures.

**Figure 2:**
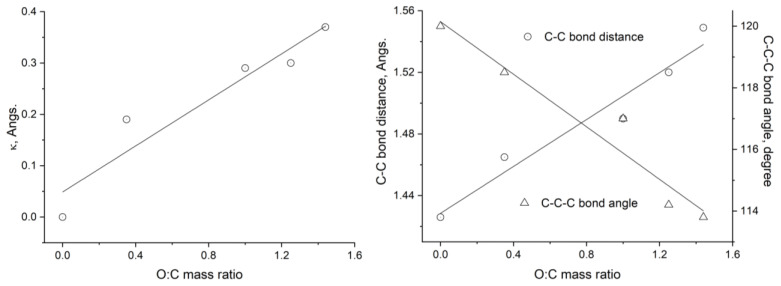
Loss of planarity (κ, **Left**) and C-C bond distances and C-C-C angles (**Right**) in the graphene framework as a function of the O/C mass ratio for graphene (Model 0) and graphene oxide derivatives (Models 1 to 4).

Geometry optimizations carried out in this work reveal the loss of planarity from graphene to GOs due to the axial C-O coordination (Figure 1 and Figure 2). This phenomenon is related to other structural changes in graphene (Figure 2): the increase in the mean C-C bond distance (from 1.426 Å in graphene Model 0 to 1.549 Å in GO Model 4) and the decrease in the C-C-C bond angles (from 120.0° in Model 0 to 113.8° in Model 4). These modifications can be rationalized considering the change in the carbon hybridization [6,8] from the sp^2^ hybridization, typical of planar conjugated systems like graphene, to the sp^3^ one, corresponding to tetrahedral carbon when oxygen is attached axially to the carbons [39]. 

Moreover, the structural changes described above are more pronounced as the O/C ratio increases (in the sequence from Model 1 to Model 4). The *molecular* structure of the GOs studied here is a combination of planar and non-planar fragments [39], which has a direct consequence on the electronic structure of the GO surface. The contribution of the planar fragments becomes less significant as the O/C ratio increases. The outlined structural changes are possible because of the flexibility of the carbon atom network that, at the same time, shows a noticeable resistance to deformation: whereas the O/C ratio rises approximately 300% from Model 1 to Model 4, the bond distance increases and the bond angles decrease, each by less than 10%. 

**Figure 3:**
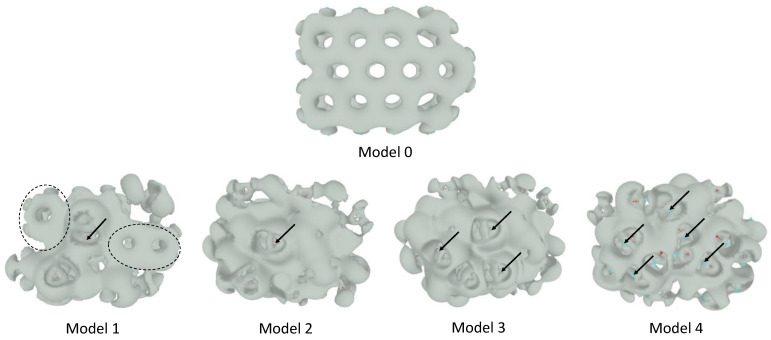
Positive electron density deformations obtained by DAM calculations for graphene (model 0) and GOs (Models 1 to 4) considered in this work. Representations correspond to a contour value of 0.001 a.u. See the text for the meaning of the arrows and the regions bounded by dashed lines.

**Figure 4:**
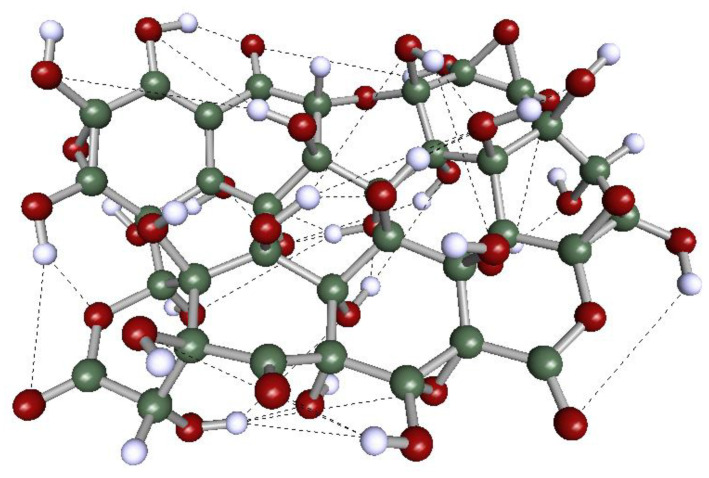
*Quasi*-two-dimensional H-bond network calculated in this work for Model 4 (GO model with O/C mass ratio = 1.45). H-bonds, represented by dashed lines, were computed for d_(O···H)_ < 2.0 Å and a_(H···O-C)_ ≤ 120°.

Positive electron density deformation, calculated by the Deformed Atoms in Molecules (DAM) methodology [40,41], in graphene (Model 0) is strongly delocalized over the entire *molecular* domain (Figure 3), showing symmetrical charge accumulations above and below the molecular plane, as is characteristic of conjugated systems [40]. This behavior is altered in GOs due to the O-C bonds (Figure 3), with their electron density deformation characterized by the following two features:

- The electron density deformations display two distinct shapes. The first shape (delimited by dotted lines in Model 1, Figure 3, for example) resembles the density deformation of graphene (Model 0). It is localized on the planar fragments of the less oxidized GOs (Models 1 and 2) and results from the contribution of the π-electron cloud of the conjugated system. The second one is located on the oxygen-substituted fragments of the *molecule*, which is a direct consequence of the non-paired, n-electrons of the oxygen atoms. The π-electron cloud remains intact in the non-substituted segments of Models 1 and 2 but practically disappears in the structure of Model 4, where the O-C substitution is the maximum evaluated in this work.
- The electron density deformations show several discontinuities (indicated by arrows in Figure 3) in the GOs studied here. These correspond to regions of the *molecular* domain where ionic-like bonds (instead of covalent ones) predominate. It arises in the presence of hydrogen atoms strongly polarized positively, involved in H-bond interactions. This phenomenon has been well recognized in DAM studies of H-bond systems. The OH group distribution on the *molecular* surface of the GOs with high O/C ratios (Model 4, for example) guarantees a *quasi*-two-dimensional network of H-bonds (Figure 4), which could support strong proton conduction in this kind of material.

Calculated Natural Population Analysis (NPA) atomic charges [42] confirm that H-bonding additionally polarizes the atoms involved. The charges are −0.5 for the oxygen atoms of epoxide, carbonyl, and ether groups not involved in H-bonds but decrease to −0.6 when they participate in hydrogen bonds. In similar situations, the charges of the oxygen atoms belonging to hydroxyl groups fall from −0.7 to −0.8. 

The electrostatic potential (calculated by the DAM theory, Figure S1) on the graphene surface (Model 0) is homogeneously negative as is expected of systems with nucleophilic character. This picture is substantially modified by oxygen substitution (Models 1 to 4). Under oxidation, some regions of the *molecular* surface exhibit positive electrostatic potential. The polarization of the surface (positive and negative potential) becomes more relevant as the O/C ratio increases, conferring amphoteric character to the GO surfaces. 

### 2.2. COSMO Analysis of the Charge Distribution on the Molecular Surface of the GOs

**Figure 5:**
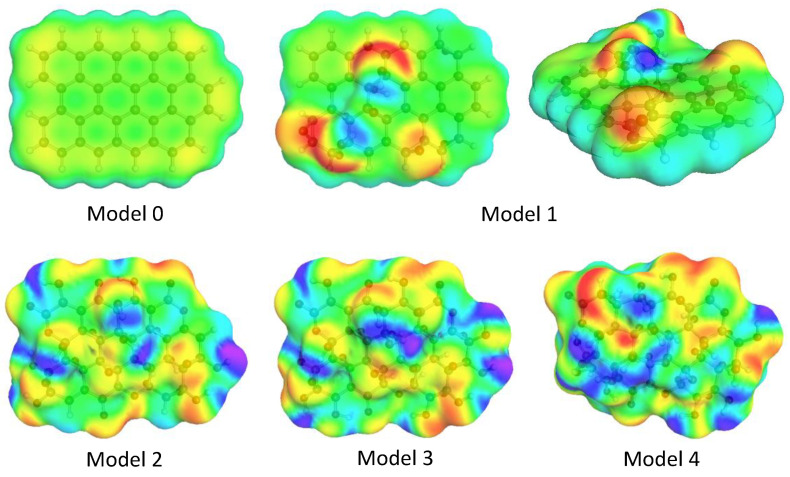
Sigma-surfaces of the graphene (Model 0) and GOs (Models 1 to 4) obtained by COSMO calculations on the optimized geometries. Color code: red and blue regions are, respectively, hydrogen bonding proton-acceptor and proton-donor molecular segments; green-yellow regions can participate in van der Waals and dispersive interactions. For Model 1, both superior and lateral views are given.

The σ-surface of graphene (Model 0) is dominated by green–yellow colors (Figure 5), revealing a highly polarizable π electronic structure, along with a slightly positively charged framework of carbon atoms (blue–green zones). These results are consistent with the electrostatic potential description offered by DAM calculations (Figure S1). 

Oxygen substitution generates new red and blue regions on the σ-surface (Figure 5), indicating the presence of groups positively and negatively polarized at the surface of the graphene oxides, which correspond to the hydrogen and oxygen atoms, respectively. Despite the polarization of the GO surface described above, yellow–green regions persist at their surfaces (Models 1 to 4) along with the red and blue regions already mentioned. Similarly to the case of graphene, this indicates the presence of neutral highly polarizable groups.

**Figure 6:**
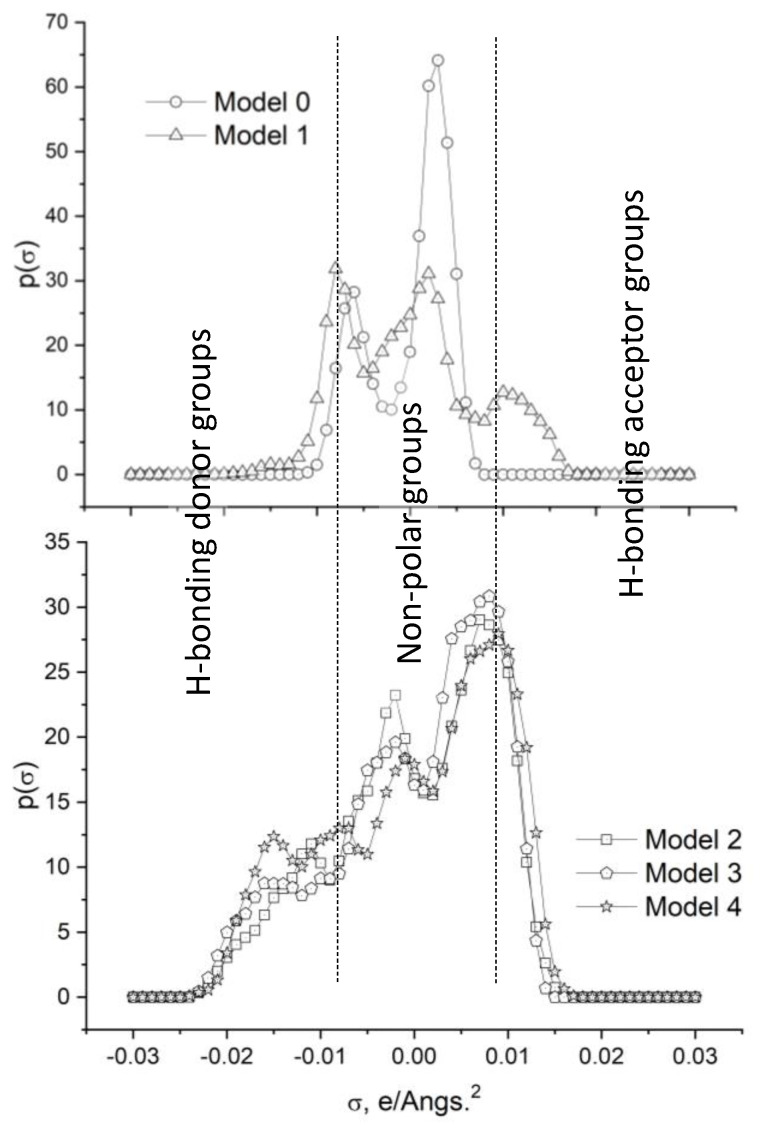
Sigma-profiles of the graphene (Model 0) and GOs (Models 1 to 4) obtained by COSMO calculations on the previously optimized geometries.

Neutral segments (green–yellow regions in Figure 5) on the *molecular* surface are reflected in the σ-profile (Figure 6) by two well-differentiated peaks in the region −0.008 < σ (e/Å^2^) < 0.008.

In the σ-profile of the GOs, a broadband arises in the regions where σ < −0.008 e/Å^2^ (HB donor region), and a *shoulder* appears where σ > 0.008 e/Å^2^ (HB acceptor region). The first of these regions is associated with the H atoms of the hydroxyl groups, whereas the second one is related to the O atoms in the different oxygenated groups attached as O-C substituents. These two types of signals in the σ-profiles suggest that the oxidized graphene derivatives could show a certain limited amphoteric character. The signal at σ ≈ −0.015 e/Å^2^ grows with the O/C ratio, peaking in Model 4, which correlates with the increasing number of the hydroxyl groups attached to the graphene surface (see Section 3.1. Molecular Models). Besides, the significant reduction of the peak at σ ≈ 0.003 e/Å^2^ due to oxygen substitution (from Model 0 to Models 1–4) reflects the break of the *molecular* conjugation by the oxygen coordination.

The polarized charge distribution on the molecular surface of the solvents and monomers considered in this work (Figure S2) consistently reflects what is well-known about the electronic structure of conventional molecular species. Octane is a non-polar solvent whereas water, ethanol, and the monomers are capable of H-bond interacting with both donor and acceptor groups, thus also showing amphoteric character. 

### 2.3. Interaction of GOs with Molecular Species of Different Polarities: Thermodynamic Features

**Figure 7:**
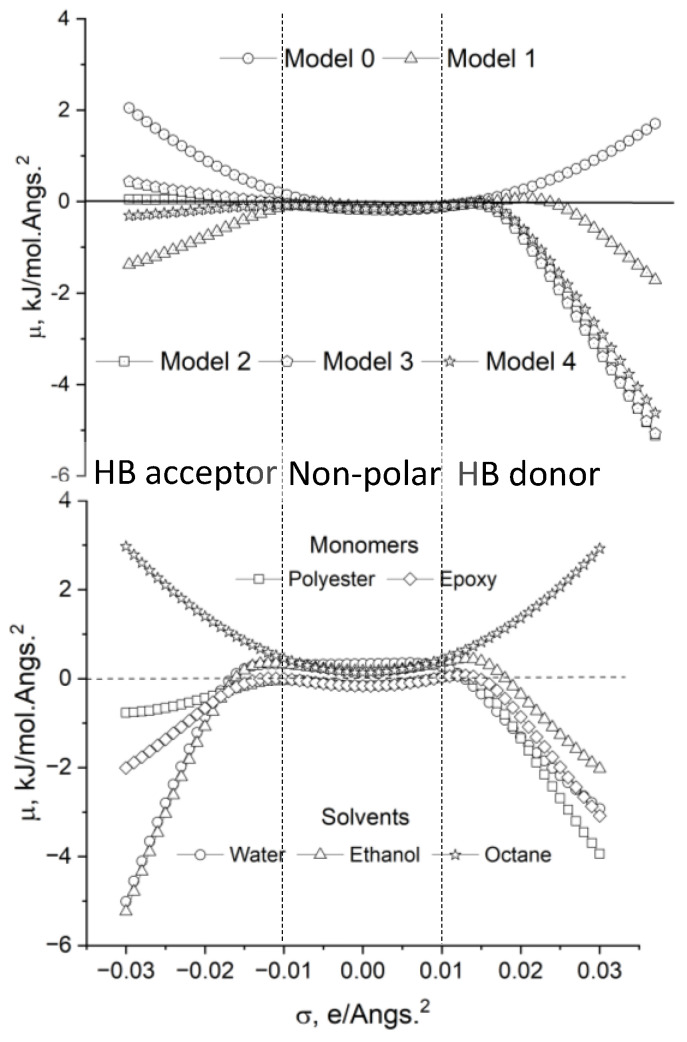
Sigma-potentials of the GOs [**Up**] and solvents and monomers [**Down**] obtained by COSMO-RS calculations on the previously optimized geometries.

Regarding the σ-potentials of graphene (Model 0) and octane (Figure 7), it can be inferred that they do not interact favorably (μ > 0 kJ/mol·Å^2^) with polar components, which exhibit μ < 0 kJ/mol·Å^2^ if σ < −0.008 e/Å^2^ and σ > 0.008 e/Å^2^. The thermodynamic behavior is noticeably different when it comes to the polar solvents and monomers and the graphene oxides selected for the current analysis (Figure 7). The polar solvents and the monomers exhibit a marked amphoteric character (μ < 0 kJ/mol·Å^2^ for σ < −0.008 e/Å^2^ and σ > 0.008 e/Å^2^) being capable of interacting favorably with both proton-donor and proton-acceptor groups, i.e., the curves μ (kJ/mol·Å^2^) vs. σ (e/Å^2^) reveal a certain symmetry. However, this symmetry is broken for some of the GOs studied. All of them show HB potential donor capacity (μ < 0 kJ/mol·Å^2^ for σ > 0.008 e/Å^2^) due to the hydrogen atoms of the hydroxyl groups, the value of μ (kJ/mol·Å^2^) being, as a rule, more negative as the number of OH groups in the GO increases (blue regions in the σ-surfaces, Figure 4). This is analogous to what occurs with the peak at σ ≈ −0.015 e/Å^2^ in the σ-profile (Figure 6), which is already discussed. However, in several cases (Models 2 to 4), μ ≈ 0 kJ/mol·Å^2^ when σ < −0.008 e/Å^2^, showing a weak acceptor capacity of the oxygen attached to the carbonous surface. This is congruent with the yellow–red color distribution on the σ-surface (Figure 5) and the presence of a *shoulder* in the σ-profile (Figure 6) when σ > 0.008 e/Å^2^, which are also mentioned before. 

*Pseudo*-liquid binary mixtures (octane + graphene/GOs) exhibit *H^Excess^* > 0 kJ/mol for practically all composition intervals and GrOs, which confirms the non-feasible thermodynamic nature of the interactions among them. In mixtures (octane + GOs (Models 2 to 4)), the van der Waals interactions are favorable to the mixture, but misfit and HB ones are the opposite. 

**Figure 8:**
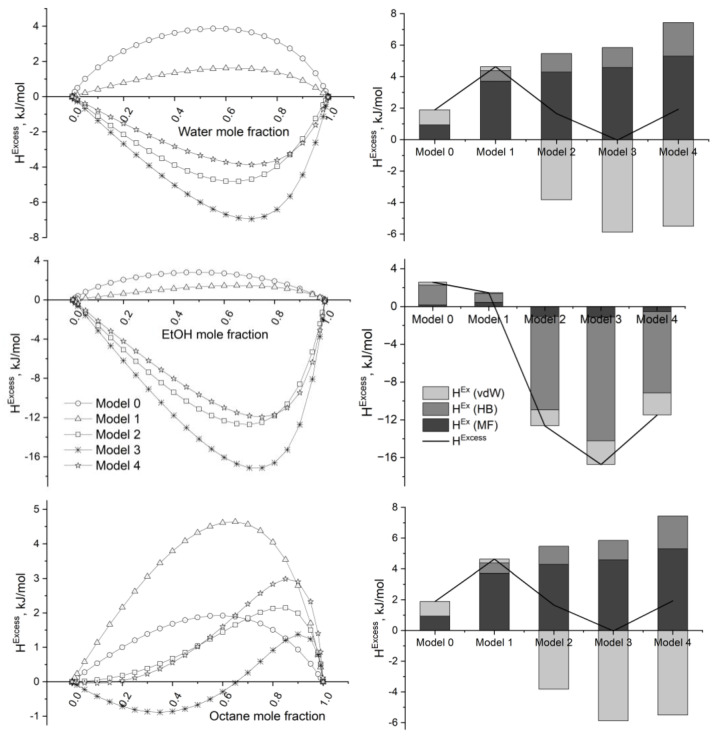
[**Left**]. Excess enthalpies (kJ/mol) obtained by COSMO-RS calculations for the binary *pseudo*-liquid mixtures (solvent + graphene/GO). [**Right**]. Decomposition of the excess enthalpy in terms of the intermolecular interaction contributions. Mixture composition = 65 mole% solvent. T = 298 K; P = 1 atm.

**Figure 9:**
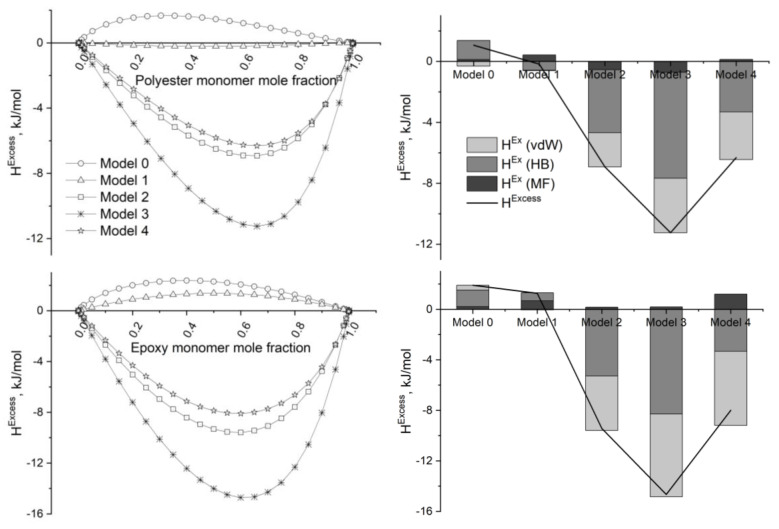
[**Left**]. Excess enthalpies (kJ/mol) obtained by COSMO-RS calculations for the binary *pseudo*-liquid mixtures (monomer + graphene/GO). [**Right**]. Decomposition of the excess enthalpy in terms of the intermolecular interaction contributions. Composition = 65 mole% monomer. T = 298 K; P = 1 atm.

Excess enthalpies (*H^Excess^*) of the *pseudo*-liquid (polar solvent/monomer + graphene/GO, where polar solvent = water and ethanol) binary mixtures evidence a similar behavior (Figure 8 and Figure 9). The interaction of the molecular species with the most oxidized GOs (Models 2 to 4) is always favorable (*H^Excess^* < 0 kJ/h) but not with graphene (Model 0) or the low-oxidized (reduced) GO (Model 1), although this last graphene derivative displays an amphoteric character (Figure 7). 

*H^Excess^* is minimum, as a rule, for mixtures containing approximately 35 mole% GO. Highly negative excess enthalpies, in the interval from −7 to −17 kJ/mol, characterize the interaction in (polar solvents/monomer + GO (Model 3)) mixtures. In addition, these interactions are predominantly of an HB nature. In fact, mean HB contribution to *H^Excess^* in (polar solvent/monomers + GO) mixtures, where polar solvent = water and ethanol and GO = Models 2 to 4, ranges from 51% to 98%, behaving as follows: water (98%) > ethanol (77%) > polyester monomer (58%) > epoxy monomer (51%). An opposite trend is observed for the van der Waals contribution to *H^Excess^* in these same mixtures: epoxy monomer (55%) > polyester monomer (38%) > ethanol (16%). Van der Waals interactions are significant in those cases where conjugated rings participate in the molecular structure, i.e., practically doubling from polyester to epoxy monomers. 

It is noteworthy that the sequence obtained for *H^Excess^* of the *pseudo*-liquid (polar solvent/monomer + GO) binary mixtures (Model 3 < Model 2 < Model 4) shows a minimum for the mixture where GO ≡ Model 3 (Figure 8 and Figure 9). This phenomenon could be explained considering that: (i) H-bonding contribution is predominant in the inter*molecular* (polar solvent/monomer + GO) interactions (Figure 8 and Figure 9), and (ii) deep oxidation of the graphene substrate favors the intra*molecular* H-bonding interaction over the inter*molecular* H-bonding interactions with polar molecules. 

The previous results should be considered when deciding the best application of a specific graphene-based material or when selecting/designing the graphene derivative with optimized properties for a specific application. For instance, deep-oxidized GOs could be recommended as proton conductors because the intra*molecular* H-bonds favor the formation of a quasi-two-dimensional proton-conducting network (Figure 4). On the contrary, applications in adsorption, catalysis, and other related operations demand GOs capable to inter*molecular* interact via H-bonding. The optimum O/C ratio for any application should be determined on a case-by-case basis. 

Quantifying the energy of the interaction between the solvents and the GOs is crucial for selecting an adequate exfoliant agent in the synthesis of graphene and graphene derivatives dispersions. To achieve this, the Hansen solubility parameters (Equation (4)), which account for the dispersive, polar, and hydrogen bonding interactions between the solvent molecule and the GO surface, are widely used [18,43,44,45,46]:

(1) $$\frac{{\Delta H}_{Mix}}{V}\approx \left[{\left(\delta_{D,S}-\delta_{D,G}\right)}^{2}+\frac{{\left(\delta_{P,S}-\delta_{P,G}\right)}^{2}}{4}+\frac{{\left(\delta_{H,S}-\delta_{H,G}\right)}^{2}}{4}\right]\cdot \theta_{G}$$

where Δ*H_Mix_* is the enthalpy of mixing of the mixture (solvent + GO); *δ_D_*, *δ_P_*, and *δ_H_* are the dispersive, polar, and hydrogen bonding components of the cohesive energy density; and *S* and *G* subscripts represent solvent and graphene, respectively. *θ_G_* is the molar fraction of graphene in the mixture.

From the results presented in this paragraph, it is evident that excess enthalpy calculated by the COSMO-RS method for (solvent + GO) *pseudo*-liquid binary mixtures could be an alternative descriptor to the Hansen solubility parameters when selecting an appropriate exfoliant for the dispersion of GOs. They both consider a complete set (dispersive van der Waals, polar, and hydrogen bonding) of interactions. 

### 2.4. A Structural Insight to the Interactions of the Polar Solvents with Low-Oxidized GOs

As previously noted, *H^Excess^* > 0 kJ/mol (Figure 8 and Figure 9) for all composition intervals in the *pseudo*-liquid binary (polar solvents/monomers + GO (Model 1)) suggests that the interaction among them is not thermodynamically favorable. This result seems to contradict the fact that both the GO (Model 1) and the polar solvents/monomers exhibit an amphoteric character (Figure 5, Figure 6 and Figure 7) due to the presence of oxygenated and hydroxyl groups in their structure. This hypothesis was confirmed by optimizing the structure of the (ethanol + Model 1) cluster at m062x/6-311G(d,p) computational level, i.e., transitioning from the continuous solvation model to the discrete molecular description of the interactions. 

The energy stabilization (Δ*E*) of the cluster along the optimization sequence was evaluated as follows:

(2) $$\Delta E={EE}_{\left(GO-EtOH\right)}-\left({EE}_{\left(GO\right)}+{EE}_{\left(EtOH\right)}\right)$$

where *EE* is the total electronic energy of the system. The term $\left({EE}_{\left(GO\right)}+{EE}_{\left(EtOH\right)}\right)$ accounts for the total energy of the individual fragments that constitute the cluster while in a non-interacting state. 

**Figure 10:**
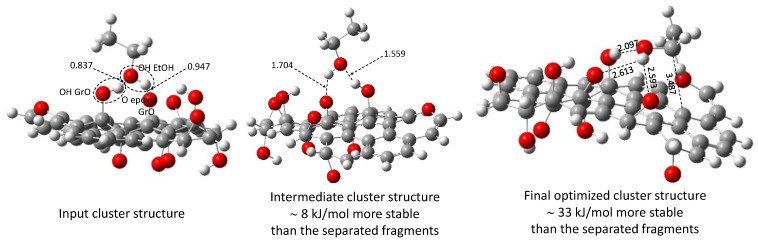
(EtOH + GO (Model 1)) cluster structures representative of the geometry optimization sequence. [**Left**]—Input geometry. [**Middle**]—Optimized structure at the first stage of the optimization pathway. [**Right**]—Final optimized structure. Interatomic distances are given in Ångstrom.

The cluster input structure (Figure 10) in the current optimization exercise was generated bearing in mind the premise of the H-bond formation. Accordingly, the ethanol molecule (EtOH) was placed perpendicularly to the GO surface, the H of its OH group pointing to the O atom of an epoxy group of the GO, and the O of the ethanol OH group facing the H of an OH group on the GO surface (Figure 10). O···H interatomic distances in the input structure were fixed below 1.0 Å (shorter than a typical H bond) to ensure higher repulsive inter-nuclear energies than those corresponding to the minimum energy structure and to the energy of the non-interacting fragments. Due to the elevated computational cost of the full optimization of the cluster, individual fragments were not optimized, i.e., their structures remained frozen during the optimization of the relative position between them. 

**Figure 11:**
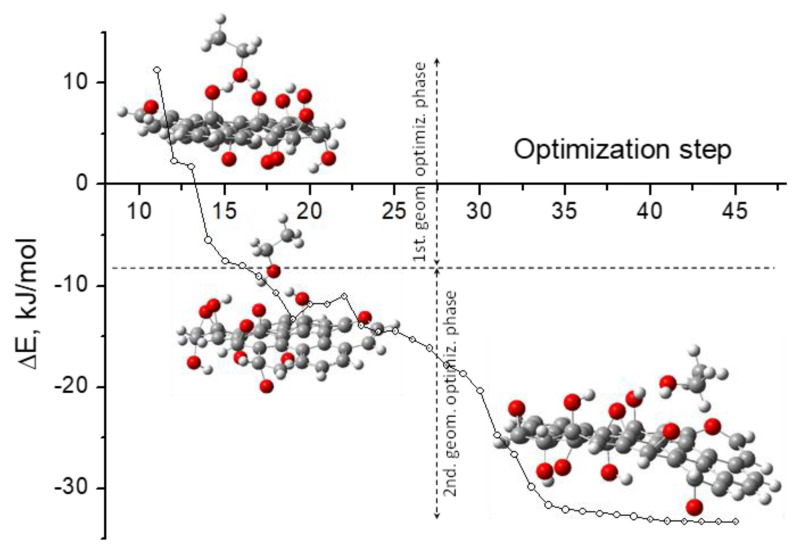
Relative values of the total electronic energy along the optimization pathway of the (ethanol + GO (Model 1)) cluster. The structures of the cluster in three optimization steps are included. For more details on the cluster structures, see Figure 10.

Although the geometry optimization was carried out uninterruptedly (i.e., in only one run), two well-differentiated phases can be recognized in the optimization profile (Figure 11). Firstly, the computational procedure optimized the O···H interatomic distances (enlarged compared to the input structure) preserving the perpendicular orientation of the ethanol molecule relative to the GO surface. 

The resulting structure of the first geometry optimization phase is nearly 8 kJ/mol more stable than the individual isolated molecules (Figure 10 and Figure 11). Here, the O···H interatomic distances (ca. 1.6 Å) are typical of H-bonding interactions. In the second phase, the EtOH molecule changed the orientation relative to the GO surface, adopting an almost parallel position relative to it but maintaining the H-bond *configuration*. The structure obtained at the end of the geometry optimization is practically 33 kJ/mol more stable than the individual isolated molecules, i.e., 76% of the stability (relative to the individual isolated molecules), as cluster aggregation is achieved in the second phase of the optimization pathway. Here, the O···H interatomic distance increases, relative to the intermediate structure, to ca. 2.5 Å, with the mean distance between the ethanol molecule and the GO surface being ca. 3.5 Å (calculated as the distance between the C(OH) of the EtOH and the closest C atom of the GO).

Thus, it can be argued that the additional stabilization reached in the second optimization phase, and the change in the orientation of the ethanol molecule relative to the GO surface are related. It seems that the orientation change has two correlated consequences. On the one hand, it makes possible the van der Waals interaction between the neutral fragments (Figure S2) of the ethanol molecule and similar ones on the GO surface (Figure 5). In fact, in Model 1, the green–yellow-colored *molecular* fragments located around H-bonding centers (red- and blue-colored fragments) are capable of interacting via the van de Waals mechanisms with similar ones in other molecules. On the other hand, the H-bonding interactions are *more comfortable* from a steric point of view (even when the O···H interatomic distance increases). The surface electron distribution in the GOs is not *planar*, as observed in both the electron density (Figure 3) and COSMO (Figure 5) calculations. H-bonding centers in Model 1 (lateral view of the σ-surface, Figure 5) are located at the top of the electron density *hills*, whereas extended van der Waals *valleys* surround them.

**Figure 12:**
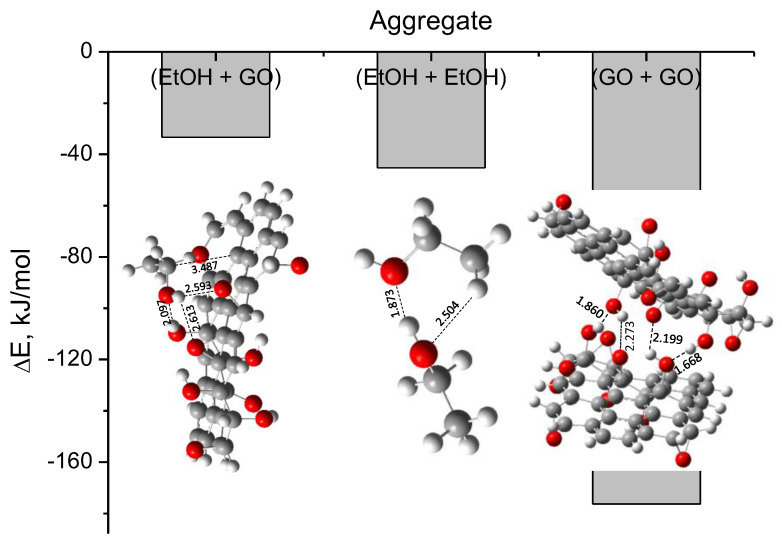
Relative energies of the aggregates (EtOH + GO), (EtOH + EtOH), and (GO + GO) relative to the individual isolated *molecular* species. GO is represented by Model 1 in the corresponding cases. Optimized structures of each cluster are also shown. Interatomic distances are given in Ångstrom.

From the set of results presented in Section 2.3 and Section 2.4, an obvious question arises: Why is the interaction (EtOH + GO (Model 1)) not thermodynamically favorable for any composition of the mixture (Figure 8) if the cluster EtOH-GO (Model 1) is energetically stable (Figure 10 and Figure 11)? The solution to this question relates to the stability of the (EtOH + EtOH) and (GO + GO) aggregates. The minimum energy structures obtained for each of them (Figure 12) are, respectively, 45 and 176 kJ/mol more stable than the individual isolated species (Figure 12). This means that (EtOH + EtOH) and (GO + GO, where GO = Model 1) are more stable than (EtOH + GO, where GO = Model 1). (EtOH + EtOH) aggregate could model the ethanol dimerization, whereas (GO + GO) dimer could refer to the aggregation of dispersed graphene oxides in the solvent.

## 3. Materials and Methods

### 3.1. Molecular Models

**Figure 13:**
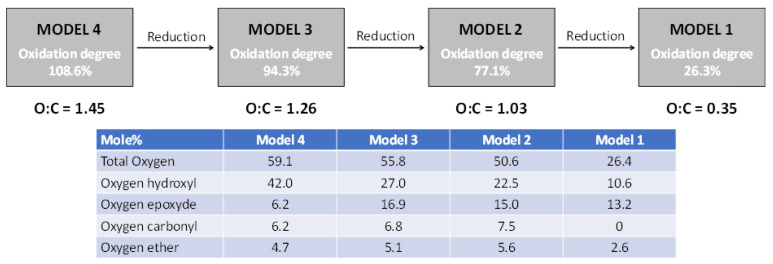
Experimental information used to define both the O/C mass ratio of the GO models and the distribution by type of the oxygenated functional groups. The O/C mass ratio was established by elemental analysis, and group distribution by IR and Raman analysis [11,12]. The oxidation degree is presented on a molar basis.

Five structures, having 38 carbon atoms (12 fused hexagons), were proposed (Figure 1) to model both the graphene (Model 0) and its oxidized derivatives (oxidized graphene, Models 1 to 4). The GO models were obtained by attaching axially oxygen atoms to some selected carbons at the graphene structure. In this way, different oxygenated groups were created: hydroxyl, epoxide, carbonyl, and ether. To model GOs of different oxidation degrees, several of these C-O bonds were created. Thus, O/C atomic ratios of 0.35 (Model 1), 1.03 (Model 2), 1.26 (Model 3), and 1.45 (Model 4) were assessed. They represent a wide interval of O/C ratios obtained experimentally (Figure 13) [12], ranging from low-oxidized (reduced) to high-oxidized GOs. Model 1 could represent a reduced (low-oxidized) GO, whereas the other models correspond to deep-oxidized GOs. 

The relative quantity of each kind of oxygenated group attached to the graphene framework also corresponds to experimental data (Figure 13) [11], but their distribution on the *molecular* domain when creating the models was somewhat arbitrarily determined. In all the structures proposed, valences of peripheral carbons were completed with hydrogens when appropriate. Although 24-fused hexagon models were also evaluated in this work, they were discarded due to the marked increase in the computational cost without a significant improvement in the results.

Ethanol, water, octane, as well as polyester and epoxy monomers (Figure S3), were selected to study the interactions of molecular solvents and monomers with the GOs. They can display both polar and non-polar interactions with graphene oxides. (GO + solvent) and (GO + monomer) interactions were analyzed because of their importance in the liquid exfoliation used to prepare GO suspensions [19,43,44]; in the catalytic activity [47] of GOs for several processes, such as the synthesis of graphene-based membranes [1] and polymers; in the adsorption of drugs and other species on the GO surface [29,30,31,32,33,34,48]; as well as in the synthesis of composite materials graphene/epoxy resin [49,50,51] with different potential applications. Dimer aggregates were proposed to study the interactions between graphene and molecular species. Three types of dimmers were evaluated: (GO + molecule), (molecule + molecule), and (GO + GO). More information on the strategy followed to define these aggregates and additional computational details on their geometry optimization are given in the next paragraph, along with the analysis of the results.

### 3.2. Quantum Chemical Calculations and Geometry Optimization

DFT methods were used in all the quantum chemical calculations. Two computational levels were employed in geometry optimization tasks. Individual structures of graphene, GO, solvents, and monomers were optimized at the B3LYP/6-31G(d,p) level. This functional gives excellent results in structure optimization and energy calculations for systems having covalent bonds, even if they are large in size. However, dimer aggregates were optimized at the m062x/6-311G(d,p) level to ensure a good description of the *long-range* interactions like dispersive, ionic, and H-bonds. In fact, m05-2x and m06-2x functionals, having a greater number of parameters than B3LYP, were developed [52] to correct the description of the non-local interactions. Additionally, polarized and diffuse atomic basis sets were selected to guarantee enough flexibility to model the *weak* interactions.

In all the geometry optimization jobs, the *molecules* were considered isolated, i.e., they do not interact with other species. The minimum energy condition for the optimized structures was confirmed by frequency calculations. Quantum chemical calculations were performed with the Gaussian 16 program package [53].

Interatomic distances and bond and torsion angles were used to characterize the molecular structures of the individual species and the aggregates. In GO, the axial coordination of oxygen atoms breaks [8,54,55] the planarity of the graphene. Planarity loss of graphene in GO was evaluated relative to the mean plane determined by the carbon atoms, the *z* coordinate of which (*z_m_*) was defined as follows:

(3) $$z_{m}=\frac{\sum_{n}z_{i}}{n}$$

where *z_i_* is the *z* Cartesian coordinate for each carbon atom, and *n* is the total number of carbon atoms in the GO (*n* = 38).

The planarity loss (*κ*) was calculated as follows:

(4) $$\kappa =\frac{\sum_{n}\left|z_{i}-z_{m}\right|}{n}$$

In the current work, *intramolecular* H-bonds at the GO surfaces were studied. Here, it is considered that the O···H interaction conforms a hydrogen bond for interatomic O···H distances around 2.0 Å and H···O-C angles up to 120°.

### 3.3. Electronic Structure

Atomic charges were used to characterize the electronic structure of the species studied. They were calculated by the Natural Population Analysis formalism as implemented in Gaussian 16 (Population = NBO keyword) [42]. 

Furthermore, electron density calculations by the Deformed Atoms in Molecules (DAM) methodology [40,41] were carried out to study the electronic structure of graphene and GOs. This method establishes a bridge between the quantum mechanical concept of electron density and the concepts of empirical chemistry throughout the electron density deformation. The molecular electron density is partitioned into minimally deformed pseudo-atomic densities, which are achieved by assigning to each atom the charge distributions centered on its nucleus plus the parts of the two-center ones nearest to it. This theory has been successfully applied to the analysis of the molecular electron structure of complex molecules and typical aromatic compounds [40,41]. DAM calculations were performed with version 2.0 of the program DAMQT [56]. Deformations of the atomic electronic density contributing to the chemical bond in the molecule (positive deformations) are graphically represented in this work. Negative electron density deformations are omitted for simplicity. Electron density deformations were calculated and represented here for a contour value of 0.001 a.u., which allows considering the density deformation of the π electron cloud in conjugated systems [40,41]. In addition, the electrostatic potential at the molecular surface was calculated for an electron density value of 0.001 a.u., as recommended by Bader et al. [57].

### 3.4. COSMO and COSMO-RS Calculations

COSMO (COnductor-like Screening MOdel) and COSMO-RS (COnductor-like Screening MOdel for Real Solvents) methodologies [35,36,37] were used to examine both the electronic structure of the species considered in this work and the interaction of GOs with molecular species of different polarity. COSMO is a continuum solvation model [58] that describes a molecule in solution through a quantum chemical calculation of the solute individual molecule with an approximate representation of the surrounding solvent as a continuum, with dielectric constant Ԑ → ∞ [35,36,37]. Interestingly, in COSMO and COSMO-RS theories, solids can be modeled as *pseudo*-liquid systems, making it possible to apply both formalisms to the analysis of the interaction between solid surfaces and molecules in operations such as adsorption [38]. 

COSMO calculations were performed on the molecular structures previously optimized at the B3LYP/6-31G(d,p) level, using the refined cavity construction algorithm (FINE) [59] implemented in the Turbomole program package v 7.0, which was accessed through the TMoleX v 4.5.2 [60]. Cosmo files (.cosmo) containing the polarized (as a result of the interaction of the individual molecules with the continuum medium) charge distribution were obtained with Turbomole and further used in COSMO-RS calculations, which were carried out with the BIOVIA COSMO*therm* version 20.0 program package [61]. BP_TZVPD_FINE_20 parametrization was used in all the COSMO-RS calculations. 

The polarized charge distribution on the *molecular* surface was represented in this work, as is typical in COSMO-based methodologies, through the sigma-surface and sigma-profile (σ-surface and σ-profile, respectively). The sigma-potential (σ-potential) was employed to thermodynamically characterize the potential capacity of interaction of the species assessed in this work. Finally, the excess enthalpies calculated by the COSMO-RS method were used to predict the thermodynamic feasibility of the interactions assessed.

In the COSMO-RS model, the excess enthalpy (*H^Excess^*) of a binary mixture is obtained by the algebraic sum of three contributions associated with electrostatic misfit (*MF*), van der Waals (*vdW*), and hydrogen bond (*HB*) intermolecular interactions (Equation (5)). The contributions of each type of these interactions to the excess enthalpies are evaluated in the current work.

(5) $$H^{Excess}=H^{Excess}\left(MF\right)+H^{Excess}\left(vdW\right)+H^{Excess}\left(HB\right)$$

## 4. Concluding Remarks

Quantum chemical geometry optimization at the B3LYP/6-31G(d,p) computational level reproduces reasonably well the changes in the molecular electronic structure of the graphene as a consequence of carbon oxidation to hydroxyl, epoxide, carbonyl, and ether groups. From the structural point of view, the loss of planarity, the increase in C-C bond distances, and the decrease in C-C-C bond angle are noticeable. These changes can be rationalized as a transition in the carbon hybridization from the sp^2^ hybridization, characteristic of conjugated systems, to the sp^3^ one due to the axial oxygen substitution. From the electronic point of view, the appearance of proton acceptor and proton donor centers on the solid surface is relevant, which can confer amphoteric character to the oxidized graphene derivatives and exceptional catalytic properties. GOs which have relatively low oxidation degree exhibit additionally neutral surface fragments, consisting of highly polarizable π electron cloud. In GOs with a high oxidation degree, the formation of a *quasi*-two-dimensional H bond network is also observed, which could be responsible for the high proton conduction of electricity. The interaction of oxidized graphene with polar components (solvents and monomers) is in general thermodynamically favorable, with the contribution of the H-bond being predominant. Furthermore, in those cases where it is advantageous, the van der Waals dispersive interactions can contribute to the stability of the (solvent/monomer + GO) complexes. Remarkably, the interaction of low-oxidized (reduced) graphene oxides with polar compounds (like conventional solvents and monomers) may not be thermodynamically favorable, despite their capacity to interact via H-bonding and dispersive forces. In these cases, the (solvent + solvent) dimerization and the GO aggregation could be thermodynamically more favorable than the (solvent/monomer + GO) interactions. The results obtained in this work show that the thermodynamic analyses supported by COSMO-RS calculations could be an interesting alternative to select/design both adequate exfoliants of graphene and GOs and graphene derivatives with optimized properties for specific applications.

## Data Availability

Data are contained in the article.

## Supplementary Materials

The following supporting information can be downloaded at https://www.mdpi.com/article/10.3390/molecules29163839/s1. Figure S1: Electrostatic potential at the molecular surface obtained by Deformed Atoms in Molecules (DAM) calculations for graphene (Model 0) and GOs (Models 1 to 4) considered in this work. Figure S2: Sigma-surfaces and sigma-profiles of the solvents and monomers obtained by COSMO calculations on optimized geometries. Figure S3: Optimized geometries (at the B3LYP/6-31G(d,p)) computational level) of the solvents and monomeric fragments used in this work to study the interactions with GO surfaces.

## References

[1] Bich Ha N., Van Hieu N. (2016). Promising applications of graphene and graphene-based nanostructures. Adv. Nat. Sci. Nanosci. Nanotechnol..

[2] Singh R.K., Kumar R., Singh D.P. (2016). Graphene oxide: Strategies for synthesis, reduction and frontier applications. RSC Adv..

[3] Bagri A., Grantab R., Medhekar N.V., Shenoy V.B. (2010). Stability and formation mechanisms of carbonyl- and hydroxyl-decorated holes in graphene oxide. J. Phys. Chem. C.

[4] Dideikin A.T., Vul A.Y. (2019). Graphene oxide and derivatives: The place in graphene family. Front. Phys..

[5] Talyzin A.V., Sundqvist B., Szabo T., Dekany I., Dmitriev V. (2009). Pressure-induced insertion of liquid alcohols into graphite oxide structure. J. Am. Chem. Soc..

[6] Pei S., Cheng H.-M. (2012). The reduction of graphene oxide. Carbon.

[7] Sun L. (2019). Structure and synthesis of graphene oxide. Chin. J. Chem. Eng..

[8] Sheka E.F., Popova N.A. (2013). Molecular theory of graphene oxide. Phys. Chem. Chem. Phys..

[9] Dreyer D.R., Park S., Bielawski C.W., Ruoff R.S. (2010). The chemistry of graphene oxide. Chem. Soc. Rev..

[10] Chua C.K., Pumera M. (2014). Chemical reduction of graphene oxide: A synthetic chemistry viewpoint. Chem. Soc. Rev..

[11] Romero A., Lavin-Lopez M.P., Sanchez-Silva L., Valverde J.L., Paton-Carrero A. (2018). Comparative study of different scalable routes to synthesize graphene oxide and reduced graphene oxide. Mater. Chem. Phys..

[12] Del Prado Lavin-Lopez M., Romero A., Garrido J., Sanchez-Silva L., Luis Valverde J. (2016). Influence of different improved hummers method modifications on the characteristics of graphite oxide in order to make a more easily scalable method. Ind. Eng. Chem. Res..

[13] Marcano D.C., Kosynkin D.V., Berlin J.M., Sinitskii A., Sun Z., Slesarev A., Alemany L.B., Lu W., Tour J.M. (2010). Improved synthesis of graphene oxide. ACS Nano.

[14] Stankovich S., Dikin D.A., Piner R.D., Kohlhaas K.A., Kleinhammes A., Jia Y., Wu Y., Nguyen S.T., Ruoff R.S. (2007). Synthesis of graphene-based nanosheets via chemical reduction of exfoliated graphite oxide. Carbon.

[15] Mkhoyan K.A., Contryman A.W., Silcox J., Stewart D.A., Eda G., Mattevi C., Miller S., Chhowalla M. (2009). Atomic and electronic structure of graphene-oxide. Nano Lett..

[16] Cai W., Piner R.D., Stadermann F.J., Park S., Shaibat M.A., Ishii Y., Yang D., Velamakanni A., An S.J., Stoller M. (2008). Synthesis and solid-state NMR structural characterization of C-13-labeled graphite oxide. Science.

[17] Mattevi C., Eda G., Agnoli S., Miller S., Mkhoyan K.A., Celik O., Mastrogiovanni D., Granozzi G., Garfunkel E., Chhowalla M. (2009). Evolution of electrical, chemical, and structural properties of transparent and conducting chemically derived graphene thin films. Adv. Funct. Mater..

[18] Hernandez Y., Lotya M., Rickard D., Bergin S.D., Coleman J.N. (2010). Measurement of multicomponent solubility parameters for graphene facilitates solvent discovery. Langmuir.

[19] Coleman J.N., Lotya M., O’Neill A., Bergin S.D., King P.J., Khan U., Young K., Gaucher A., De S., Smith R.J. (2011). Two-dimensional nanosheets produced by liquid exfoliation of layered materials. Science.

[20] Elhaes H., Abdel-Salam A.I., Gomaa I., Ibrahim A., Yahia I.S., Zahran H.Y., Ezzat H.A., Zahran M., Abdel-wahab M.S., Refaat A. (2023). Facile synthesis, structural, morphological and electronic investigation of Mn_2_O_3_ nano-rice shape and Mn_2_O_3_-rGO hybrid nanocomposite. Opt. Quantum Electron..

[21] Jia R., Nong X.-M., Lu H.-Q., Xiong Y.-S., Wei W., Qin W.-H., Li W. (2024). Multidimensional decipherment of interactions in invert sugar-amino acid co-degradation colorants (IACDCs) capture by polyamine co-modified shaddock peel cellulose/graphene oxide aerogel. Sep. Purif. Technol..

[22] Tahir A., Liaqat F., Saleem M., Shaik M.R., Adil S.F., Siddiqui M.R.H., Khan M. (2023). Eco-friendly synthesis of anti-microbial and anti-fungal binary metal oxide decorated reduced graphene oxide nanocomposites with complimenting density functional studies. J. Saudi Chem. Soc..

[23] Lahaye R.J.W.E., Jeong H.K., Park C.Y., Lee Y.H. (2009). Density functional theory study of graphite oxide for different oxidation levels. Phys. Rev. B.

[24] Yan J.A., Xian L.D., Chou M.Y. (2009). Structural and electronic properties of oxidized graphene. Phys. Rev. Lett..

[25] Yan J.A., Chou M.Y. (2010). Oxidation functional groups on graphene: Structural and electronic properties. Phys. Rev. B.

[26] Bagri A., Mattevi C., Acik M., Chabal Y.J., Chhowalla M., Shenoy V.B. (2010). Structural evolution during the reduction of chemically derived graphene oxide. Nat. Chem..

[27] Botello-Mendez A.R., Dubois S.M.M., Lherbier A., Charlier J.-C. (2014). Achievements of DFT for the investigation of graphene-related nanostructures. Acc. Chem. Res..

[28] Demianenko E., Sencha-Hlevatska K., Sementsov Y., Kartel M. (2023). Quantum-chemical investigation of the superoxide radical scavenging by graphene oxide surface. Low Temp. Phys..

[29] Araujo W.S., Rego C.R.C., Guedes-Sobrinho D., Dias A.C., do Couto I.R., Bordin J.R., de Matos C.F., Piotrowski M. (2024). Quantum simulations and experimental insights into glyphosate adsorption using graphene-based nanomaterials. ACS Appl. Mater. Interfaces.

[30] Wu Y., Zhang X., Liu C., Tian L., Zhang Y., Zhu M., Qiao W., Wu J., Yan S., Zhang H. (2024). Adsorption behaviors and mechanism of phenol and catechol in wastewater by magnetic graphene oxides: A comprehensive study based on adsorption experiments, mathematical models, and molecular simulations. ACS Omega.

[31] Tri N.N., Ho D.Q., Bao N.T.G., Trung N.T. (2024). The adsorption of tetracycline, ciprofloxacin on reduced graphene oxide surfaces: Role of intermolecular interaction. Chem. Phys..

[32] Murmu M., Huda, Mobin M., Aslam R., Banerjee P. (2024). Adsorption of L-proline nitrate modified graphene oxide on iron surface: Density functional theory and Monte Carlo simulation study. Comput. Mater. Sci..

[33] Berisha A. (2023). Density functional theory and quantum mechanics studies of 2D carbon nanostructures (graphene and graphene oxide) for lenalidomide anticancer drug delivery. Comput. Theor. Chem..

[34] Adekoya O.C., Adekoya G.J., Sadiku R.E., Hamam Y., Ray S.S. (2022). Density Functional Theory interaction study of a polyethylene glycol-based nanocomposite with cephalexin drug for the elimination of wound infection. ACS Omega.

[35] Klamt A. (2018). The COSMO and COSMO-RS solvation models. Wiley Interdiscip. Rev. Comput. Mol. Sci..

[36] Klamt A. (2005). COSMO-RS: From Quantum Chemistry to Fluid Phase Thermodynamics and Drug Design.

[37] Klamt A. (1995). Conductor-like screening model for real solvents: A new approach to the quantitative calculation of solvation phenomena. J. Phys. Chem..

[38] Palomar J., Lemus J., Gilarranz M.A., Rodriguez J.J. (2009). Adsorption of ionic liquids from aqueous effluents by activated carbon. Carbon.

[39] He H.Y., Klinowski J., Forster M., Lerf A. (1998). A new structural model for graphite oxide. Chem. Phys. Lett..

[40] Rico J.F., Lopez R., Ema I., Ramifrez G. (2005). Chemical notions from the electron density. J. Chem. Theory Comput..

[41] Rico J.F., Lopez R., Ema I., Ramirez G. (2004). Electrostatic potentials and fields from density expansions of deformed atoms in molecules. J. Comput. Chem..

[42] Reed A.E., Weinhold F. (1985). Natural localized molecular orbitals. J. Chem. Phys..

[43] Coleman J.N. (2013). Liquid exfoliation of defect-free graphene. Acc. Chem. Res..

[44] Liu W.-W., Xia B.-Y., Wang X.-X., Wang J.-N. (2012). Exfoliation and dispersion of graphene in ethanol-water mixtures. Front. Mater. Sci..

[45] Salavagione H.J., Sherwood J., De Bruyn M., Budarin V.L., Ellis G.J., Clark J.H., Shuttleworth P.S. (2017). Identification of high performance solvents for the sustainable processing of graphene. Green Chem..

[46] Hansen C.M. (2007). Hansen Solubility Parameters: A User’s Handbook.

[47] Cui Y., Lee Y.H., Yang J.W. (2017). Impact of carboxyl groups in graphene oxide on chemoselective alcohol oxidation with ultra-low carbocatalyst loading. Sci. Rep..

[48] Moradi S., Taran M., Mohajeri P., Sadrjavadi K., Sarrami F., Karton A., Shahlaei M. (2018). Study of dual encapsulation possibility of hydrophobic and hydrophilic drugs into a nanocarrier based on bio-polymer coated graphene oxide using density functional theory, molecular dynamics simulation and experimental methods. J. Mol. Liq..

[49] Silva A.A., Stein R., Campos D., Indrusiak T., Soares B.G., Barra G.M.O. (2019). Conducting materials based on epoxy/graphene nanoplatelet composites with microwave absorbing properties: Effect of the processing conditions and ionic liquid. Front. Mater..

[50] Long J., Li S., Liang B., Wang Z. (2019). Investigation of thermal behaviour and mechanical property of the functionalised graphene oxide/epoxy resin nanocomposites. Plast. Rubber Compos..

[51] Hu G., Zhang X., Liu L., Weng L. (2019). Improvement of graphene oxide/epoxy resin adhesive properties through interface modification. High Perform. Polym..

[52] Zhao Y., Schultz N.E., Truhlar D.G. (2006). Design of density functionals by combining the method of constraint satisfaction with parametrization for thermochemistry, thermochemical kinetics, and noncovalent interactions. J. Chem. Theory Comput..

[53] Frisch M.J., Trucks G.W., Schlegel H.B., Scuseria G.E., Robb M.A., Cheeseman J.R., Scalmani G., Barone V., Petersson G.A., Nakatsuji H. (2016). Gaussian 16 Rev. C.01.

[54] Hasanzade Z., Raissi H. (2018). Density functional theory calculations and molecular dynamics simulations of the adsorption of ellipticine anticancancer drug on graphenc oxide surface in aqueous medium as well as under controlled pH conditions. J. Mol. Liq..

[55] Hasanzade Z., Raissi H. (2017). Solvent/co-solvent effects on the electronic properties and adsorption mechanism of anticancer drug thioguanine on graphene oxide surface as a nanocarrier: Density functional theory investigation and a molecular dynamics. Appl. Surf. Sci..

[56] Lopez R., Fernandez Rico J., Ramirez G., Ema I., Zorrilla D. (2015). DAMQT 2.0: A new version of the DAMQT package for the analysis of electron density in molecules. Comput. Phys. Commun..

[57] Bader R.F.W., Carroll M.T., Cheeseman J.R., Chang C. (1987). Properties of atoms in molecules: Atomic volumes. J. Am. Chem. Soc..

[58] Tomasi J., Persico M. (1994). Molecular interactions in solution: An overview of methods based on continuous distribution of the solvent. Chem. Rev..

[59] Klamt A., Diedenhofen M. (2018). A refined cavity construction algorithm for the conductor-like screening model. J. Comput. Chem..

[60] Steffen C., Thomas K., Huniar U., Hellweg A., Rubner O., Schroer A. (2010). Software news and updates TmoleX. A graphical user interface for TURBOMOLE. J. Comput. Chem..

[61] Eckert F., Klamt A., Koch L. (2019). COSMOThermX.

