# Supplementary material for: An Insight into the Molecular Electronic Structure of Graphene Oxides and Their Interactions with Molecules of Different Polarities Using Quantum Chemical and COSMO-RS Calculations
Source: Molecules. 2024 Aug 13;29(16):3839. doi: 10.3390/molecules29163839 (PMC11357623; doi:10.3390/molecules29163839)
Supplement: Supplementary file 1 [file molecules-29-03839-s001.zip › molecules-3146258-supplementary/SupplementaryMaterial.pdf]

An Insight into the Molecular Electronic Structure of Graphene Oxides and Their Interactions with Molecules of Different Polarities Using Quantum Chemical and COSMO-RS Calculations

Víctor R. Ferro [1,*], Sonia Merino [2], Rafael Lopez [3] and José L. Valverde [2]

[1] Departamento de Ingeniería Química, Universidad Autónoma de Madrid, 28049 Madrid, Spain
[2] Departamento de Ingeniería Química, Universidad de Castilla la Mancha, 13091 Ciudad Real, Spain;
soniamefe@gmail.com (S.M.); joseluis.valverde@uclm.es (J.L.V.)
[3] Departamento de Química Física Aplicada, Universidad Autónoma de Madrid, 28049 Madrid, Spain;
rafael.lopez@uam.es
* Correspondence: victor.ferro@uam.es

Supplementary material

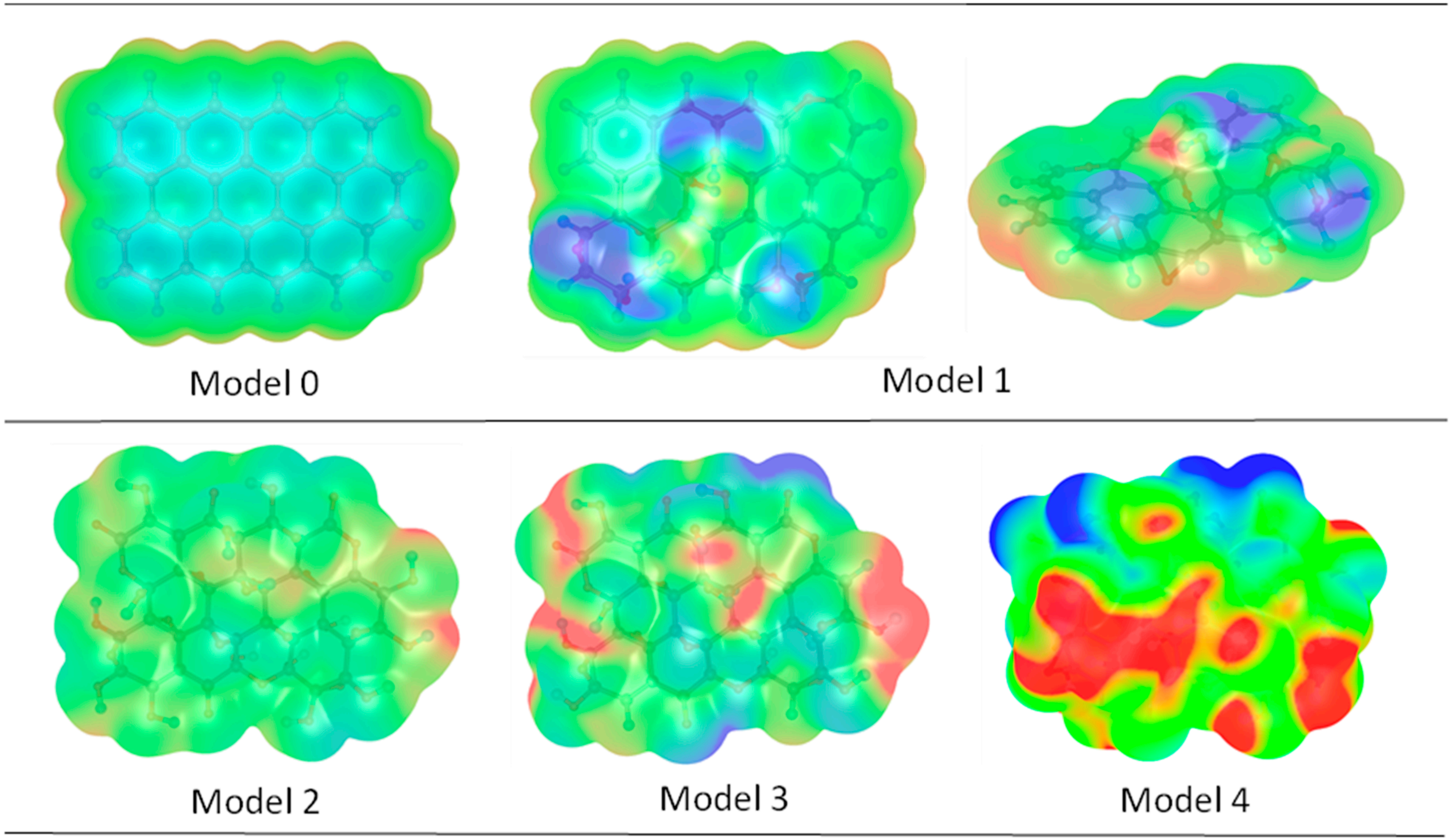

Figure S1. Electrostatic potential at the molecular surface obtained by Deformed Atoms in Molecules (DAM) calculations for graphene (Model 0) and GOs (Models 1 to 4) considered in this work. Representations correspond to an electron density value of 0.001 a.u. Color code: blue - negative potential, red - positive potential. Green-yellow regions of the surface correspond to non- or low- polarized *molecular* fragments. In the current calculations the *molecules* are considered isolated *i.e.* they do not interact with other species.

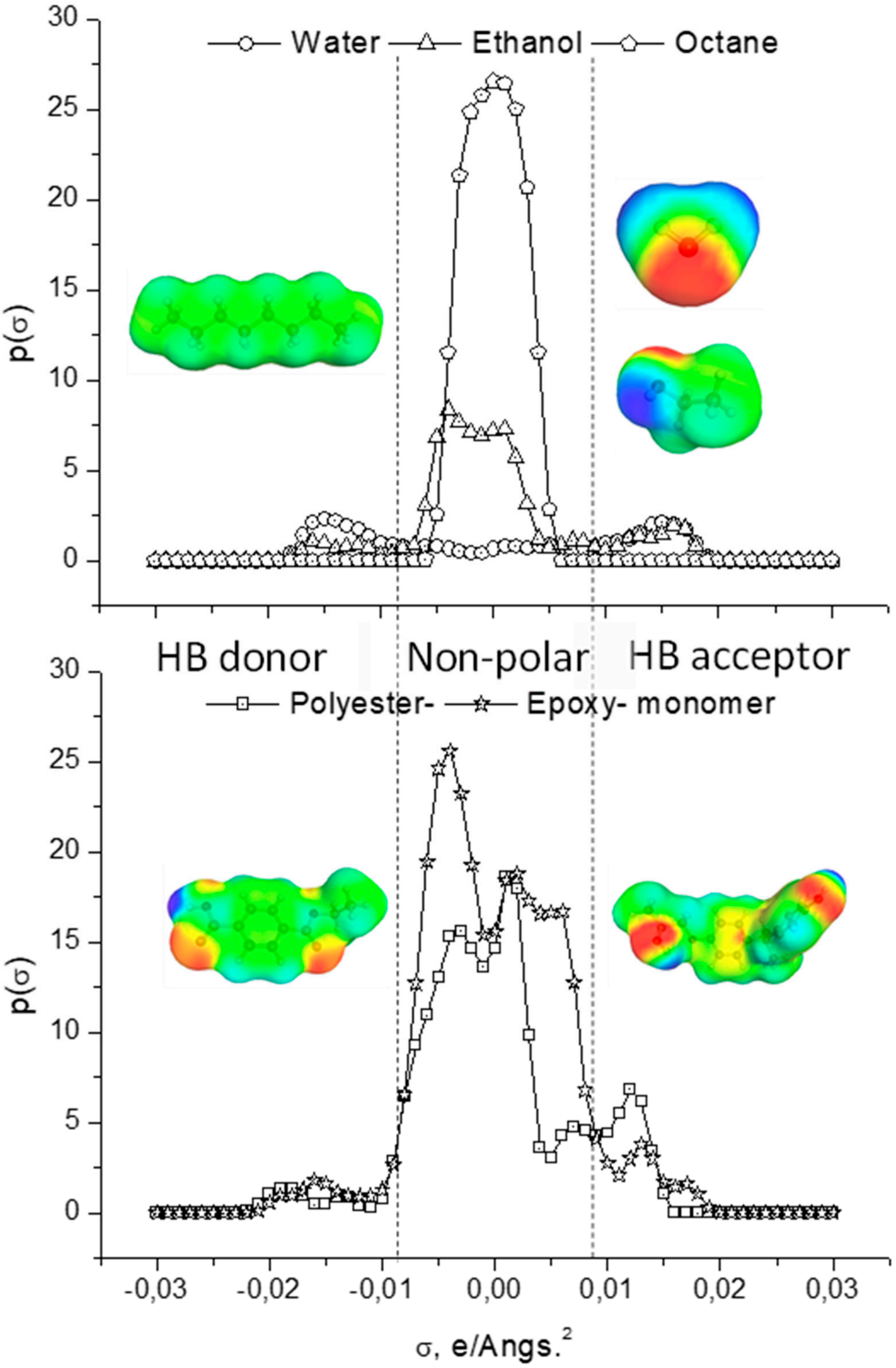

Figure S2. Sigma-surfaces and sigma-profiles of the solvents (Up) and monomer (Down) obtained by COSMO calculations on the optimized geometries. Color code for the sigma-surfaces: red and blue regions are, respectively, hydrogen bonding proton-acceptor and proton-donor molecular segments; green-yellow regions can participate in van der Waals and dispersive interactions.

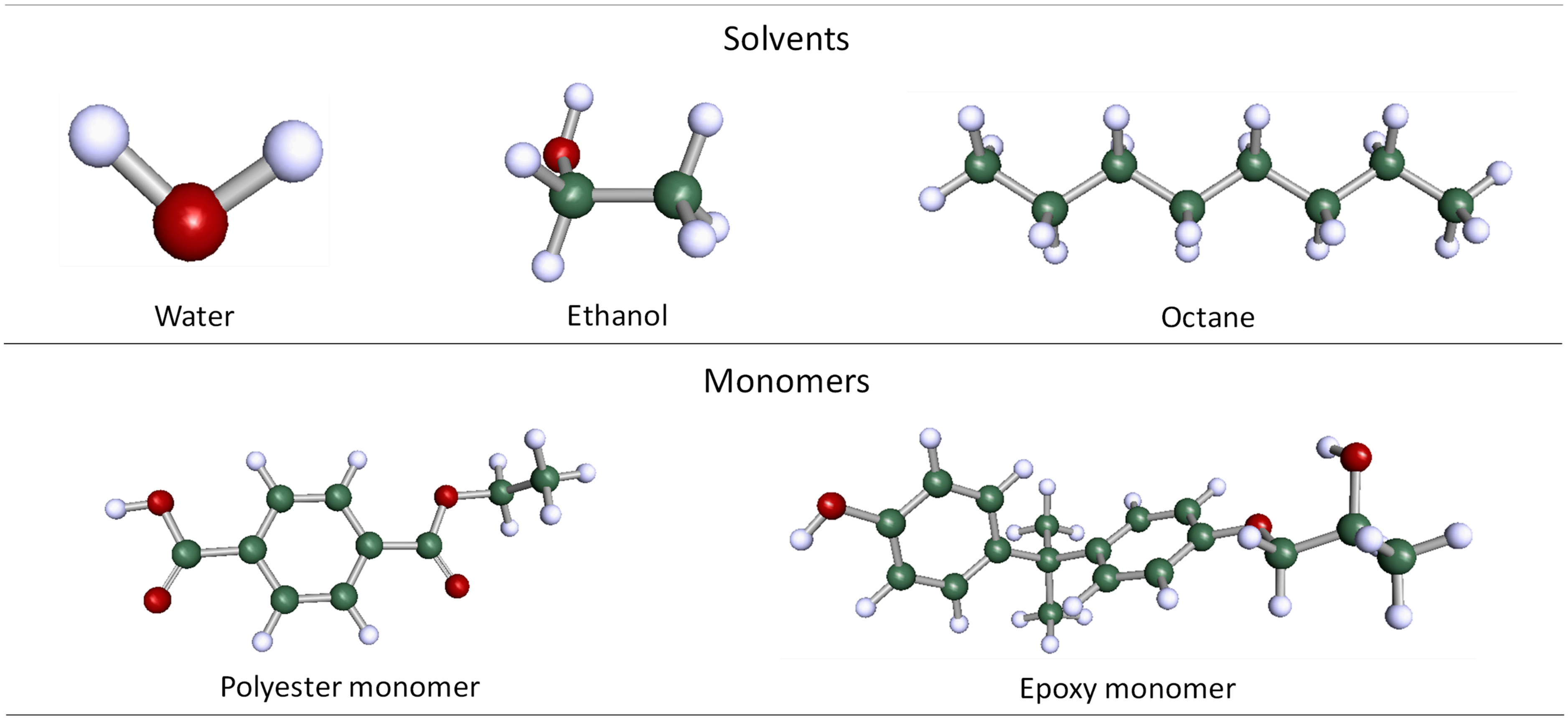

Figure S3. Optimized geometries (at B3LYP/6-31G(d,p)) computational level) of the solvents and monomeric fragments used in this work to study the interactions with GOs surfaces. Atom color code: dark grey - carbon, red - oxygen, white - hydrogen. For simplicity only one structure is displayed for each compound. Nevertheless, in COSMO-RS calculations different conformers were considered when corresponded.
